# Blue Light Deprivation Produces Depression-Like Responses in Mongolian Gerbils

**DOI:** 10.3389/fpsyt.2020.00233

**Published:** 2020-04-08

**Authors:** Hong Hu, Chenping Kang, Xiaohong Hou, Qi Zhang, Qinghe Meng, Jianjun Jiang, Weidong Hao

**Affiliations:** ^1^ Department of Toxicology, School of Public Health, Peking University, Beijing, China; ^2^ Beijing Key Laboratory of Toxicological Research and Risk Assessment for Food Safety, Beijing, China

**Keywords:** blue light, depression, melatonin, HPA axis, gerbils

## Abstract

**Background:**

Depression is a leading cause of disability worldwide and is a major contributor to the overall global burden of disease, but its etiology is poorly understood. It has been reported that a disrupted biological rhythm, in terms of a shortened light duration and total darkness, can cause depression-like behaviors in animals. Blue light was reported to have an inhibitory effect on melatonin, which is considered an important clock rhythm biomarker. In the present study, we investigated the effects of blue light deprivation on depressive-like behaviors in gerbils and explored the underlying mechanisms.

**Methods:**

Gerbils were housed under white light with a filter to block the blue light or without a filter. The behaviors of the gerbils were observed. The biological rhythm, 5-HT, hypothalamic-pituitary-adrenal (HPA) axis and melanopsin pathway were analyzed.

**Results:**

We found that blue light deprivation (BLD) induced depression-like behavior in gerbils. Melatonin lost its rhythm, and corticosterone (CORT) levels decreased in the morning in the BLD group. Lower corticotropin-releasing hormone (CRH) in the hypothalamus and lower adrenocorticotropin hormone (ACTH)/CORT in serum were observed after BLD. Furthermore, 5-HT in the serum and brain were decreased after BLD. Additionally, BLD affected the blue light sensitivity protein melanopsin and its pathway, with downregulation of the proteins melanopsin, PKCα, and c-Fos and the mRNA levels of *c-fos* and *trpc3* and upregulation of the protein p-PKCα.

**Conclusions:**

Our findings indicated that BLD might produce depression-like behaviors in gerbils. Melatonin arrhythmicity, HPA axis abnormalities, 5-HT decreases and melanopsin pathway changes might be associated with the depression behavioral phenotype in gerbils.

## Introduction

Depression is a leading cause of disability worldwide and is a major contributor to the overall global burden of disease (1). The cause of depression has been complex and insufficiently understood to date, posing a challenge to its proper treatment. Increasing evidence has shown that light deficiency or deprivation causes both depressive symptoms in humans and a depressive phenotype in rodents (2–5). Seasonal affective disorder (SAD) patients show atypical symptoms of depression in the fall and winter, and these symptoms are relieved in the spring or summer. The occurrence and remission of SAD suggest that light plays an important role in depression. In clinical practice, light treatment has been used as one of the methods to relieve depression in SAD patients. However, there are few reports on which band of the light spectrum affects depression (6, 7). Moreover, to attenuate the harmful effect of blue light on the retina, the blue-filtered intraocular lens was used. However, compared with the blue-filtered intraocular lens, a conventional unstained intraocular lens that produces more blue light has been shown to improve sleep and quality of life and relieve depression in older adults (8). These results suggested that blue light may have positive effects on depression. Also, studies have observed higher melatonin levels in depression patients and approximately 80% have sleep disturbances in SAD patients (9, 10). Circadian disturbances have also been observed in depression patients in psychological and physiological domains (11, 12). Melatonin is produced by the pineal gland in a circadian rhythm with peak levels observed at night in human beings (13, 14). It is closely related to sleep and is generally considered a clock rhythm biomarker (15–17). Compare with other visible light, blue light is the most effective for melatonin inhibition and could reset melatonin circadian rhythm (15). We speculated that lack of blue light could had an effect on depression resulting high melatonin level. Earlier study shows that melatonin may be a significant factor in depression-like behaviors in gerbils (18). Additionally, there are a group of retinal ganglion cells called ipRGCs prolonged to the circadian pacemaker, suprachiasmatic nucleus (SCN). The ipRGCs contain melanopsin, which is involved in the regulation of serum melatonin level, sleep regulation and biological circadian rhythm (19, 20). The ipRGCs and melanopsin are also sensitive to blue light (21, 22). In summary, we hypothesized that blue light had an effect on depression.

A link between abnormalities of the hypothalamic-pituitary-adrenal (HPA) axis and depression has been one of the most consistently reported findings in psychiatry, and HPA-axis hyperactivity was a distinct feature of persons with melancholic depression (23, 24). Corticosterone (CORT) is synthesized in the adrenal gland in response to adrenocorticotropin hormone (ACTH) released from the anterior pituitary, which is under the control of corticotropin releasing hormone (CRH) secretion from the paraventricular nucleus (PVN) of the hypothalamus controlled by the SCN. There are multilevel interactions between the HPA axis and the circadian system (e.g., melatonin) (25). Melatonin has been found to prevent the adrenal response to ACTH (26). It results that the high levels of melatonin inhibits the secretion of CORT (27). Consistently, there is a lack of CORT during the day in some depression patients (24). CORT is secreted in a circadian rhythm influenced by light and characterized by low and high levels at the start of the inactive and active phases, respectively (28, 29). Considering these findings, we speculated that the lack of CORT might be related to the increased melatonin secretion caused by a lack of blue light.

Another accepted hypothesis of depression is the monoamine hypothesis. Studies show decreased 5-HT levels in depression patients (30), and increased 5-HT bioavailability in synapses has been shown to effectively relieve depressive symptoms (30, 31). Some antidepressants work by elevating synaptic 5-HT concentrations. Tricyclic clomipramine remains effective along with serotonin-noradrenaline reuptake inhibitors (SNRIs) and selective serotonin reuptake inhibitors (SSRIs), suggesting that 5-HT plays an important role in depression (32, 33). 5-HT, as the metabolite source of melatonin, maybe also have a relationship with blue light.

Our earlier study showed that blue light deprivation (BLD) could produce depression-like behaviors in nocturnal rats (34). Compared with nocturnal animals, diurnal animals may be more sensitive to light deprivation and more homologous to humans in the context of light and circadian rhythms (35, 36). Diurnal animals have become an advantageous model for affective disorders (36). Bilu showed that blue light could ameliorate depression-like behavior in diurnal animals (37). It is not known, however, whether the BLD affects the circadian rhythm and mood in diurnal animals. Therefore, in the present study, we used diurnal gerbils to evaluate the effect of blue light deprivation on depression-like behaviors and the biological rhythm of melatonin and CORT, and we also studied the possible mechanism through the HPA axis, 5-HT and melanopsin pathway in the retina.

## Materials and Methods

### Animals

Male Mongolian gerbils (*Meriones unguiculatus*) weighing 31.3 ± 4.6 g (5~6 weeks) were obtained from the Zhejiang Province Laboratory Animal Center, China. Two gerbils in every cage were housed in transparent (polycarbonate) cages to allow social contact and avoid extreme sensory deprivation. These cages had a controlled temperature (24 ± 1°C), a relative humidity of 50–70% and controlled noise (less than 60 dB (A)). The animals had a 12:12 light:darkness cycle (lights on at 07:00 h, geographical Beijing time) and free access to water and standard diet food (Vital River Laboratory Technology Co., Ltd., China). The current study was consistent with all institutional and national guidelines and the Laboratory Animals Guidelines of Welfare and Ethics Statement and was approved by the Animal Care and Use Committee of Peking University (LA2017227).

### Light Conditions

**Figure 1:**
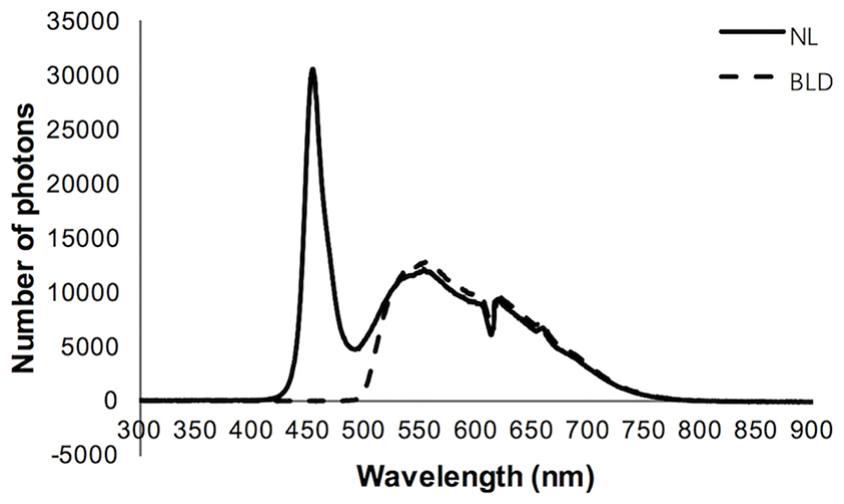
Characteristics of the light spectrum used in the study.

A light-emitting diode (LED) light source was used. The white LED light source without or with a filter was applied to the experiment, namely, the normal light (NL) or blue light deprivation (BLD) group, respectively. The cut-off band of the filter is below 490 nm (Daheng Optics, China). The two light source spectra were detected by a spectrometer (**Figure 1**) (Avantes, Netherlands). The spectra were approximately 420-760 nm for NL and 490-760 nm for BLD. The 16 LED beads were distributed evenly on a plate to ensure equal light intensity distribution. According to the Laboratory Animal Requirements of Environment and Housing Facilities (GB 14925-2010, China), the illumination intensity in the cages with NL was 199.0 ± 2.8 Lx, and the irradiance was 7.52 ± 0.13 mW/cm^2^. The illumination intensity and the irradiance of the BLD group were 256.7 ± 7.6 Lx and 7.45 ± 0.20 mW/cm^2,^ respectively, to avoid an energy difference with NL. The illumination intensity and irradiance were detected by a Spectral Irradiance Colorimeter (Everfine, China). The illumination intensity during the dark period was 0 Lx.

### Experimental Design

After one week for adaptation, animals were randomly assigned to the groups by a body weight randomization method and treated with NL or BLD. The body weight and food consumption were determined weekly. Blood (100 μL) was taken from the jugular vein every four weeks with isoflurane anesthesia. The tail suspension test (TST) and forced swimming test (FST) were conducted every four weeks, and the first tests were conducted at the 5^th^ week. The FST was conducted two days after the TST to reduce the interaction between the two behavior tests. To measure melatonin and CORT, we collected blood from the jugular with isoflurane anesthesia five days after the depression-like behaviors occurred. We collected blood serum at 01:00 and 13:00 on day 1, 04:00 and 16:00 on day 2, 07:00 and 19:00 on day 4, 10:00 and 22:00 on day 5. Three days after blood was taken from the jugular, the animals were sacrificed, and the blood samples were collected after isoflurane anesthesia. The brain and retinal tissues were collected.

### Behavioral Testing

The depression-like behaviors of the gerbils were assayed using the tail suspension test and forced swimming test. The TST and FST were performed between 08:00 and 12:00 under approximately 10 Lx white light.

#### Tail Suspension Test

The TST has been widely used for testing “behavioral despair of mouse” (38). The TST in gerbils was previously verified (39). In brief, the animal held by the tail, with hollow cylinders around the base of the tail of the gerbils to prevent tail-climbing behavior, was suspended 50 cm from the floor, and the behavior of the animal was recorded by a video camera for 6 min. Then, the immobility time of the last 4 min (indicator of depression-like behavior) was assessed by a blinded method.

#### Forced Swimming Test

FST is used to assess the depression-like behavior of rats, which is termed learned helplessness. FST was performed as described previously (40, 41) with some modifications to accommodate gerbils. Briefly, the gerbils were individually placed for 6 min in a transparent tank (length: 60 cm, width: 40 cm, height: 40 cm) containing water (25 ± 1°C) to a depth of 30 cm. The immobility time (no active movement or floating with only small activities to keep the animal from drowning) for the 6 min of the test was scored from the videotapes by an observer who was blinded to the treatment conditions of the gerbils.

### Frozen Sections of Brain Tissue

The gerbils were anaesthetized with isoflurane, the limbs were fixed and the chest was cut open to fully expose the heart. With gentle clamping of the left ventricle by hand, a tipped intravenous injection needle was carefully inserted into the left ventricle and fixed with tweezers. At this time, blood pulsation was observed in the intravenous injection needle. After the right auricle was cut, the animals were first perfused with 0.9% NaCl for 4 min to remove the blood, followed by perfusion of 4% paraformaldehyde (PFA) in 0.1 M phosphate buffer for approximately 10 min, which resulted in a muscle tremor throughout the whole body. The brain tissue was completely separated and fixed in 4% PFA fixative solution overnight at 4°C. After the end of postfixation, the brains were cryoprotected at 4°C in 30% sucrose in PBS until the tissues sank to the bottom of the solution (approximately one week). Then, the brain tissue was embedded with OCT and stored at -80°C until testing.

### Enzyme-Linked Immunosorbent Assays for Serum Determinations

The melatonin, 5-HT, CORT and ACTH levels in the serum were determined according to the procedure of the manufacturer (Elabscience, China). Briefly, 50 μL of standard or sample was added to each well, and 50 μL of biotinylated detection Ab was immediately added to each well. Next, the plate was incubated for 45 min at 37°C and washed 3 times. Then, 100 μL of HRP conjugate was added to each well, and the plate was incubated for 30 min at 37°C and washed 5 times. Then, 90 μL of substrate reagent was added, and the plate was incubated for 15 min at 37°C. Finally, 50 μL of Stop Solution was added, and the absorbance at 450 nm was immediately determined. The results were calculated.

### Immunofluorescence for 5-HT at the Dorsal Raphe Nucleus (DR)

The locations of the dorsal raphe nuclei were determined by referring to the stereotaxic map of the gerbil brain (42). The brain tissues with the nuclei were cut into 40 μm thick sections according to the coronal direction in a freezing microtome, and the tissue sections were placed into a 24-well plate with PBS. The tissues were permeabilized with 0.1% (v/v) Triton X‐100 for 30 min. After three washes with PBS, the tissues were blocked with 3% (w/v) bovine serum albumin for 1 h at room temperature and then incubated with antibodies against 5-HT (Sigma, USA) and 3% (w/v) bovine serum albumin overnight at 4°C. After the tissues were washed with PBS, they were incubated with secondary antibodies tagged with Alexa 594 (Abcam, USA) for 1 h at room temperature in the dark. After the tissue section were washed with PBS, they were sealed with anti-fluorescence attenuation laminating agent (containing DAPI). The images were captured using a Nikon A1 confocal microscope with a 10× objective. The region of interest (ROI) was selected for every slice, and the fluorescence intensity was calculated.

### Real-Time Fluorescent Quantitative Polymerase Chain Reaction

Total RNA was extracted from the right retina using an RNA extraction kit (TransGen Biotech, China). RNA isolation was performed according to the reagent protocol instructions. The RNA concentrations were measured using a microspectrophotometer (Implen GmbH, Germany). Single-stranded cDNAs were prepared from 0.3 μg of total RNA using a PrimeScript^®^ reverse transcription polymerase chain reaction (reverse transcription‐PCR) kit (TransGen Biotech, China). A PCR mixture containing 2 μL of cDNA was prepared using SYBR^®^ Premix Ex TaqTM PCR (TransGen Biotech). All primers (**Table 1**; AuGCT, China) showed good specificity (a single peak melting curve) in the annealing temperatures determined by optimizing experiments. iQ5 (Bio‐Rad Laboratories, USA) was used for quantitative PCRs according to the manufacturer’s instructions. The relative expression of mRNA was measured by the 2−ΔΔCT method.

**Table 1:** Primer sequences for *β-actin*, OPN4, *c-fos* and *trpc3*.

Gene name	Sequences (5′-3′)
*β-actin*	Forward: TCTTGGGAGTGGGGGTGGCTTAC Reverse: TTGGCGCTTTTGACTCAGGATTTA
OPN4	Forward: CTGGAGTGCCTACGTTCCTG Reverse: GTAGCAGCCGATGATGACGA
*c-fos*	Forward: TCGTCGACCGGGGCTTACTC Reverse: AACTGCTCTACCTTGCCCCTTCTG
*trpc3*	Forward: GCCAAACATCACGGTCATCG Reverse: ACAGCTGCACGATGTACTCC

### Western Blot Analysis

Total proteins were extracted with 200 μL of RIPA per retina (1% v/v phenylmethylsulfonyl fluoride). The protein concentrations were measured with an Enhanced BCA protein assay kit (Thermo Fisher, USA). Then, the proteins were denatured in sodium dodecyl sulfate sample buffer. Protein samples (25 μg) were loaded on 8% sodium dodecyl sulfate‐polyacrylamide gel electrophoresis gels and transferred to nitrocellulose membranes (Millipore, Ireland). The membranes were blocked with 5% (w/v) skim milk, washed three times with Tris‐buffered saline and Tween 20, and then incubated with primary antibodies against melanopsin (Thermo Fisher, USA), TPH2/DDC/MAOA/PKAα/PKCα (CST, USA), β‐actin (CST, USA), CRH/p-PKCα (T497) (Abcam, UK) and c-Fos (Abcam, UK) overnight at 4°C. The membranes were incubated with secondary antibody for 1 h at room temperature after washing and then covered with ECL chemiluminescent fluid. The bands were detected by a chemiluminescence analyzer (Beijing Yuanpinghao Biotech Co., Ltd., Beijing, China).

### Statistical Analysis

The results are presented as the mean ± standard error of the mean (SEM). The body weight, rhythm of CORT and melatonin were evaluated by repeated measurement variance analysis. The other differences were evaluated by unpaired Student’s t-tests using SPSS 20. Statistical significance was assumed when *p* < 0.05.

## Results

### Blue Light Deprivation Caused Depression-Like Behaviors

**Figure 2:**
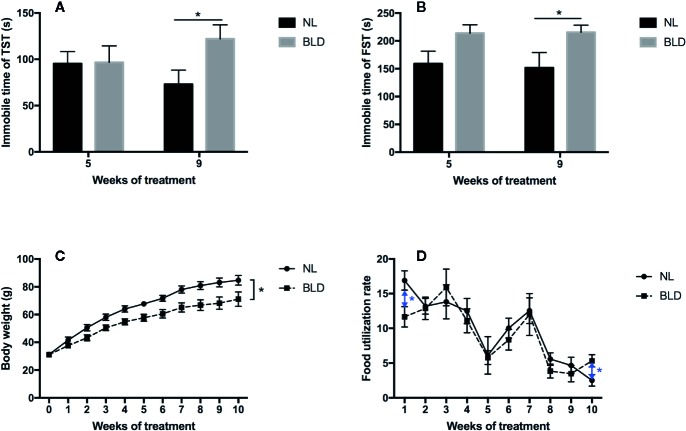
Effect of BLD on depression behaviors (**A**, TST; **B**, FST), body weight **(C)**, and food utilization rate **(D)**. (n**(A–C)** =16, n**(D)**=8, *p < 0.05 compared to NL, including black and blue).

After 8 weeks of BLD, the animals showed a clear increase in the immobility time in the TST (*p*=0.029, t=2.291, df=30) and FST (*p*=0.042, t=2.122, df=30) (**Figures 2A, B**). The body weight was decreased in the BLD group (**Figure 2C**, *p*=0.026, F=5.476, df=1), and the food utilization rate had a significant decrease in the first week (*p*=0.022, t=2.571, df=14) and increased at the 10^th^ week (*p*=0.038, t=2.296, df=14) (**Figure 2D**).

### Blue Light Deprivation Caused Low CORT and Arrhythmicity (Melatonin)

**Figure 3:**
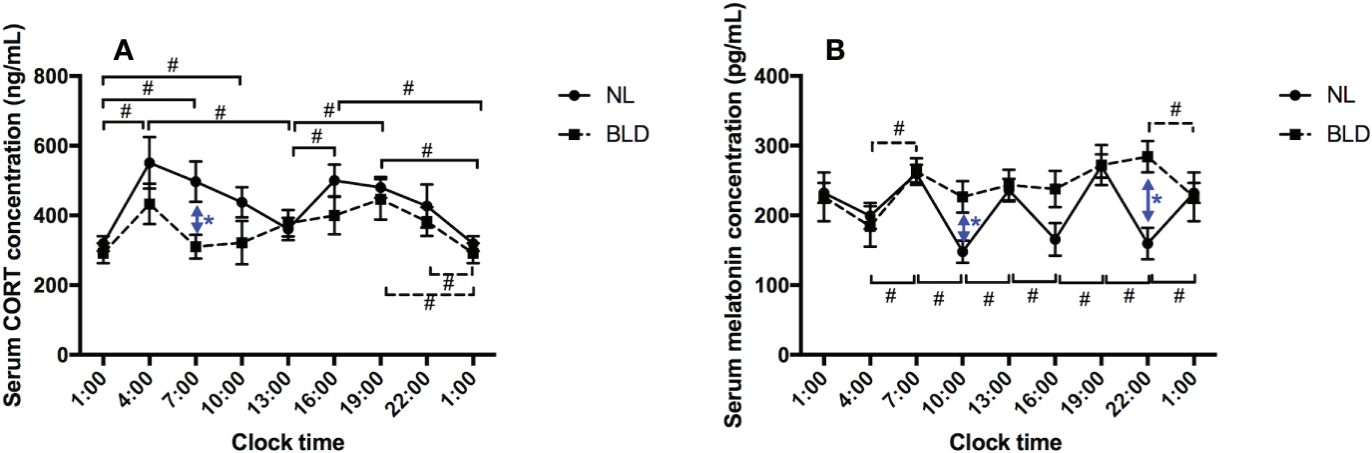
The effects of BLD on the rhythms of CORT **(A)** and melatonin **(B)**. (n(NL)=6, n(BLD)=5, *p < 0.05 compared to NL, including black and blue; ^#^ *p* < 0.05 compared between time points, solid/dotted line represents NL/BLD).

After 9 weeks of BLD, the gerbils showed a lower serum CORT concentration than that of the NL group in the morning at 07:00 (**Figure 3A**, *p*=0.036, F=9.723, df=1). The CORT concentration showed a double peak at 24 h with a 12-h cycle rhythm, and the lowest values were at 01:00 and 13:00, which were the midpoints of the light switch on and off in NL (**Figure 3A**). However, CORT lost the 12-h rhythm in the BLD group (**Figure 3A**). The animals showed a clear loss of the melatonin rhythms after BLD (**Figure 3B**). The melatonin level was higher than that in the NL group at 10:00 (*p*=0.0179, t=2.889, df=9) and 22:00 (*p*=0.0040, t=3.837, df=9) (**Figure 3B**). There were no significant differences in the melatonin levels between every time point in the BLD group. The *p*/t/df values between every time point are shown in the **Supplementary Data**.

### Blue Light Deprivation Caused HPA Axis Abnormalities

**Figure 4:**
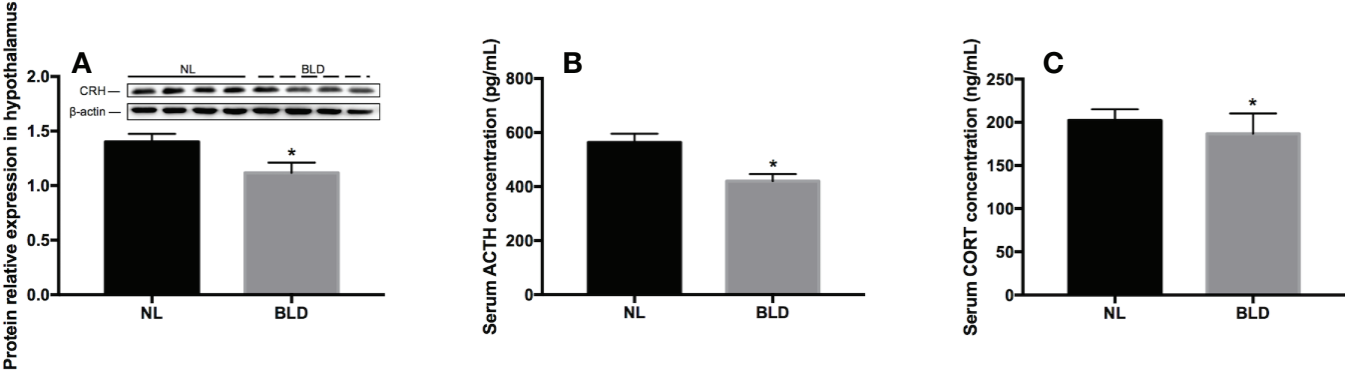
The effect of BLD on the secretion of hormones from the HPA axis. A, CRH; B, ACTH; C, CORT. (n**(A)**=8, n**(B**, **C)**=10, *p < 0.05 compared to NL. CRH, corticotropin-releasing hormone; ACTH, adrenocorticotropic hormone; CORT, corticosterone).

In the animals, after 10 weeks of BLD, the CRH secreted by the hypothalamus (**Figure 4A**, *p*=0.003, t=3.515, df=14) and the ACTH secreted by the pituitary gland were lower than those in the NL group (**Figure 4B**, *p*=0.002, t=3.633, df=18). The CORT secreted by adrenal glands was decreased (**Figure 4C**, *p*=0.023, t=2.484, df=18).

### Blue Light Deprivation Decreased the Neurotransmitter 5-HT in the Serum and Dorsal Raphe Nucleus (DR)

**Figure 5:**
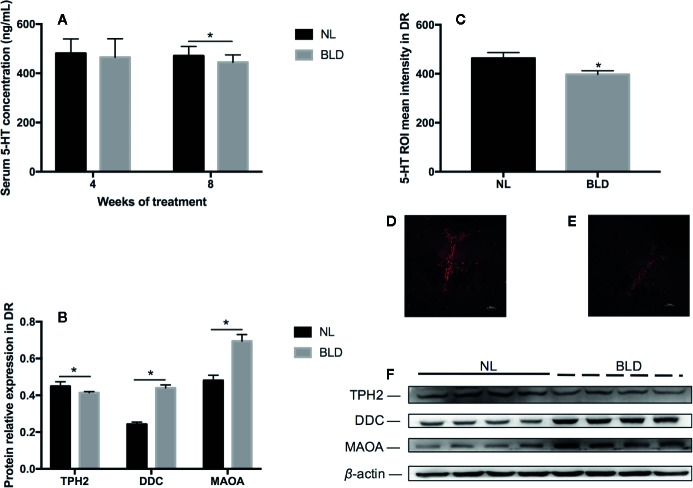
The effect of BLD on the neurotransmitter 5-HT in the serum **(A)** and DR **(C)**. **(D, E)** are representative pictures of DR 5-HT in the NL and BLD groups; B, the relative protein levels of 5-HT metabolic enzymes in the DR; **(F)**, the protein levels of TPH2, DDC and MAOA. (n**(A)**=10, n**(B)**=8, n**(C)**=6, *p < 0.05 compared to NL. DR, dorsal raphe nucleus).

In the animals, after 8 weeks of BLD, the serum 5-HT was decreased (**Figure 5A**, *p*=0.034, t=2.289, df=18), and the DA level was not changed (data not shown). In the brain, the fluorescence intensity of 5-HT in the dorsal raphe nucleus was also decreased (**Figures 5C–E**, *p*=0.038, t=2.395, df=10). Tryptophan is catalyzed by tryptophan hydroxylase 2 (TPH2) to produce 5-hydroxytryptophan in the brain (43) and then catalyzed to 5-hydroxytryptophan by 5-hydroxytryptophan decarboxylase (DDC). TPH2 is the critical enzyme for the synthesis of 5-HT. *In vivo*, serotonin can be catalyzed by monoamine oxidase (MAOA) to generate 5-hydroxytryptophan and 5-hydroxyindoleacetic acid. The changes in 5-HT metabolic enzymes in the DR included TPH2 downregulation (*p*=0.046, t=2.51, df=6) and DDC (*p* < 0.001, t=9.867, df=6) and MAOA (*p*=0.003, t=4.733, df=6) upregulation (**Figures 5B, F**).

### Blue Light Deprivation Affected the Melanopsin Pathway in the Retina

**Figure 6:**
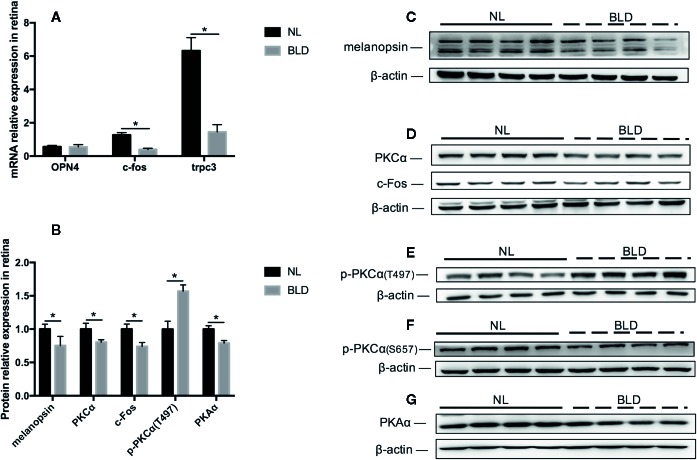
The effect of BLD on the melanopsin pathway in the retina. **(A)**, Relative mRNA expression of OPN4, *c-fos* and *trpc3*; **(B)**, Relative protein expression of the melanopsin pathway in the retina; **(C**–**G)**, Protein expression of melanopsin, PKCα/p-PKCα, c-Fos and PKAα. (n=8, *p < 0.05 compared to NL. OPN4, the mRNA of melanopsin.)

BLD caused the melanopsin protein level in the retina to decrease (*p*=0.031, t=7.363, df=14), while its mRNA OPN4 was not changed (**Figures 6A–C**). The PKCα (*p*=0.007, t=3.147, df=14), c-Fos (*p*=0.001, t=3.148, df=14) and PKAα (*p* < 0.001, t=4.984, df=14) protein levels were downregulated (**Figures 6B, D-G**). PKCζ, PKCδ and TRPC3 were not changed (data not shown). The mRNA levels of *c-fos* (*p* < 0.001, t=7.363, df=14) and *trpc3* (*p* < 0.001, t=5.829, df=14) were decreased (**Figure 6A**). p-PKCα (T497) was upregulated (*p* < 0.001, t=5.715, df=14) (**Figures 6B, E**).

## Discussion

Studies have shown that both low blue light and high white light could improve depressive symptoms in patients (7), suggesting that blue light has an effect on depression. Total darkness and shortened light duration can cause depression-like behaviors in animals, and the effects were stronger for diurnal rodents than nocturnal rodents (5, 36, 44). The energy from light might be a factor causing depression-like behaviors in animals. Given these factors, photopic lux, the standard unit of illuminance, might be inappropriate in our study. A previous study also showed that lux was inappropriate when quantifying the photic drive required for the circadian pacemaker (15). We therefore employed radiation intensity to quantify the energy of light so that the animals received the same light energy to determine whether the impact of light on depression was related to wavelength rather than energy in our study.

The melanopsin-containing ipRGCs, which are blue light sensitive, prolonged to SCN to modulate circadian rhythm. Blue light (440–480 nm) is important in non-image forming functions, including photoallodynia, sleep disorders, anxiety and depression, which are mediated by melanopsin-expressing ipRGCs (45). The present study showed that BLD for 8 weeks caused a series of profound changes in melatonin rhythms, CORT increases in the morning, the neurotransmitter 5-HT, the HPA axis and the blue light photosensitive protein melanopsin. These changes might be associated with depressive behavior. BLD might cause changes in 5-HT and the HPA axis though ipRGCs to the DR and hypothalamus pathways.

After exposure to BLD for four weeks, the body weights of the gerbils were significantly decreased. This finding was inconsistent with the results of a rat experiment in our earlier study, in which the body weight was unchanged (34). This discrepancy may be caused by the different living habits of these two species. Rats are nocturnal, while gerbils are diurnal. Diurnal gerbils might be more sensitive to BLD than rats. Studies have indicated that models of depression induced by chronic mild stress show a decrease in body weight (46, 47). The food utilization rate was decreased only in the first week, and the lack of change in later weeks may be a result of adaptation.

Our study showed that the CORT in BLD was lower than that in NL in the morning. Although the increased CORT in depression is well known, delayed morning awakening in SAD patients was also found (48). CORT is a known factor that entrains peripheral clocks, and the concentration of CORT circulating in the blood displays a dynamic circadian rhythm. The 12 h CORT rhythm in gerbils is not the same as that in humans, which is a 24 h rhythm (49). Weinert showed that without a running wheel, the daily activity pattern of gerbils was bimodal: one peak of activity occurred in the first half of the light time, and the other occurred around the light–dark transition (50). The body temperature rhythm also showed two peaks (50). The CORT results obtained in our study were consistent with the activity and temperature in Weinert’s study. Accordingly, the BLD did not change the rhythm of CORT. However, it did change the melatonin rhythm.

Melatonin is secreted at night by the pineal gland in humans. This molecule is controlled by the SCN of the hypothalamus through ipRGCs (51), and the rhythm of melatonin is generally considered to be 24 h in humans (52). Here, we reported that the melatonin of the gerbils exposed to BLD did not reset or delay but seemed to lose the rhythm, while that in NL displayed a 6 h circadian rhythm. Furthermore, the 6 h melatonin rhythm was different from the 12 h CORT rhythm. It seemed that there were two melatonin cycles corresponding to one CORT cycle. In fact, it was found that the rhythm of body temperature and urine output differed from the rhythm of sleep in humans (53), suggesting that there may be more than one regulatory mechanism for biological rhythm. CORT and melatonin might be regulated by two different rhythm systems. However, the exact mechanisms underlying these differences and similarities in CORT and melatonin in gerbils in our study are unclear.

A series of meta-analyses indicated that the presence and type of HPA axis abnormalities might vary in various subtypes of depression (54, 55). Hyperactivity of the HPA axis mainly occurs in melancholic depression, while HPA hypoactivity mainly occurs in atypical depression (24, 56). Namely, CORT is usually increased in melancholic patients but decreased in atypical patients. However, whether such a dichotomy exists is unclear In our study, BLD caused HPA in a hypoactive situation, which might be caused by the high level of melatonin with disinhibition in BLD.

The immobility time of the FST was correlated with 5-HT status (57). Patients with depression usually show low 5-HT levels (58–60). Additionally, brain 5-HT deficiency increases stress vulnerability and impairs antidepressant responses following psychosocial stress (61). Consistent with these results, we found that the gerbils, which showed some depression-like features caused by BLD, had substantially decreased 5-HT levels and an increased immobility time.

Melanopsin is a blue light-sensitive protein (15). Photoactivation of melanopsin in retinal ipRGCs activates the Gq/11 protein, which then activates the PLC-β4 and TRPC3/6/7 pathways (62). Activated PLC catalyzes the degradation of PIP2 to phosphatidylinositol diphosphate (IP3) and diacyglycerol (DAG), second signaling molecules, to generate PKC activation and Ca^2+^ influx (62, 63). Once activated, PKC transmits signals to the nucleus *via* different signal transduction pathways to activate c-Fos expression (64). c-Fos and c-Jun combine with AP-1 to promote a series of other cellular processes (64). Our results showed that the protein levels of melanopsin, PKCα, c-Fos, and PKAα and the mRNA levels of *trpc3* and *c-fos* were downregulated under deprivation of blue light. p-PKCα (T497) was upregulated. Studies have shown that p-PKCα (T497) was increased in diabetic retinas (65) and cells from myocardial infarction samples with decreased PKCα (66), and it was speculated that the increase in p-PKCα (T497) was involved in hypoxia-related signaling pathways. Therefore, we speculated that BLD might also induce hypoxia-related signaling pathways in the retina. Rhodopsin and cryptochromes are blue light-sensitive photoreceptors in addition to melanopsin (67). Therefore, the increased p-PKCα (T497) might be due to pathways other than the melanopsin pathway. A previous report showed that the presence of both dephosphorylation and phosphorylation of melanopsin depended on illumination (68), and PKA was a possible player in melanopsin phosphorylation (69). The decreased PKA in our study suggested that the BLD might also have an effect on melanopsin phosphorylation.

Our results indicated that blue light might be required for normal melatonin rhythm, 5-HT metabolism, HPA axis function and maintenance of their related behavioral roles in mood. Further studies are needed to determine the threshold of blue light deprivation for developing such abnormalities and whether subsequent white light or antidepressants could reverse these deficits. Considering that women show higher levels of depression than men (70, 71), female gerbils would be an important supplement for our study in the future.

## Data Availability Statement

The datasets generated for this study are available on request to the corresponding author.

## Ethics Statement

The animal study was reviewed and approved by the Laboratory Animals-Guideline of Welfare and Ethics Statement Animal Care and Use Committee of Peking University.

## Author Contributions

WH, QM and JJ designed the study. HH, CK, HX and QZ acquired the data, which HH and HX analyzed. HH wrote the article, which all authors reviewed and approved for publication.

## Funding

This work was supported by the Beijing Municipal Natural Science Foundation [No. 7182083].

## Conflict of Interest

The authors declare that the research was conducted in the absence of any commercial or financial relationships that could be construed as a potential conflict of interest.
